# Therapeutic effects of EGF-modified curcumin/chitosan nano-spray on wound healing

**DOI:** 10.1093/rb/rbab009

**Published:** 2021-03-13

**Authors:** Yue Li, QingQing Leng, XianLun Pang, Huan Shi, YanLin Liu, SuSu Xiao, Ling Zhao, Ping Zhou, ShaoZhi Fu

**Affiliations:** 1 Department of Oncology, The Affiliated Hospital of Southwest Medical University, Luzhou 646000, China; 2 Health Management Center, Traditional Chinese Medicine Hospital Affiliated to Southwest Medical University, Luzhou 646000, China; 3 Department of Pharmaceutics, School of Pharmacy of Southwest Medical University, Luzhou 646000, China; 4 Department of Radiology, The Affiliated Hospital of Southwest Medical University, Luzhou 646000, China

**Keywords:** curcumin, chitosan, EGF, skin regeneration, wound healing

## Abstract

Dermal injury, including trauma, surgical incisions, and burns, remain the most prevalent socio-economical health care issue in the clinic. Nanomedicine represents a reliable administration strategy that can promote the healing of skin lesions, but the lack of effective drug delivery methods can limit its effectiveness. In this study, we developed a novel nano-drug delivery system to treat skin defects through spraying. We prepared curcumin-loaded chitosan nanoparticles modified with epidermal growth factor (EGF) to develop an aqueous EGF-modified spray (EGF@CCN) for the treatment of dermal wounds. *In vitro* assays showed that the EGF@CCN displayed low cytotoxicity, and that curcumin was continuously and slowly released from the EGF@CCN. *In vivo* efficacy on wound healing was then evaluated using full-thickness dermal defect models in Wistar rats, showing that the EGF@CCN had significant advantages in promoting wound healing. On day 12 post-operation, skin defects in the rats of the EGF@CCN group were almost completely restored. These effects were related to the activity of curcumin and EGF on skin healing, and the high compatibility of the nano formulation. We therefore conclude that the prepared nano-scaled EGF@CCN spray represents a promising strategy for the treatment of dermal wounds.

## Introduction

Wound repair remains a research hotspot in the field of skin regeneration and tissue engineering. Skin is the greatest physical barrier against external forces and objects [1], injuries to which can occur as a result of tumor resection, diabetic ulcers and accidental injuries [2, 3]. Skin injuries require urgent treatment and can increase the likelihood of infection, disability and death. Moreover, if the wound is left untreated and allowed to completely heal, the potential risk of wound infection increases as the exposed subcutaneous tissue is susceptible to microbial colonization and proliferation [4, 5]. The stages of wound healing are complex and include hemostasis, inflammation, proliferation and remodeling [6–8]. Delays to healing increase the requirement for additional treatment measures. During wound management, patients require changes to dressings to avoid infection, the risk of which remains until the skin recovers its barrier function. In response to chronic trauma, a variety of damage, including stress, poor blood flow and edema, can hinder healing in the absence of aggressive treatment.

In clinical practice, the most common strategy to treat large skin wounds is to transplant autologous or allogeneic skin tissue into the wound site to compensate for tissue loss [9]. The use of wound dressings to promote skin repair and reconstruction at the defect sites offers an alternative strategy. The use of autologous or allogeneic transplantation is limited by the occurrence of secondary trauma, the low availability of donors and high cost. Using wound dressings to promote skin healing has been widely studied in terms of fiber membranes and hydrogels [10–12]. Even though fibrous mats have obvious advantages in preventing bacterial reproduction and improving air permeability, they can easily dislodge from the wound site. As an alternative strategy, soft hydrogels can fill irregular wounds, but limit gas exchange at the wound site [13]. New formulations for wound dressings are therefore urgently required to meet the above requirements.

Traditional therapies for skin defects use compounds of plant and animal origin to promote wound healing. Curcumin (CUR) is a bioactive ingredient of turmeric, displaying a variety of anti-inflammatory, anti-infection and anti-oxidation properties, with low levels of toxicity. CUR can increase the production of fibroblasts, promote the formation of granulation tissue, and accelerate the contraction and epithelialization of wounds [14, 15]. However, as CUR is hydrophobic, its bioavailability is low [16] and carriers are required to improve its stability and solubility. Many chemically synthesized polymers or natural polysaccharides have been used to deliver CUR to treat skin defects [9, 17, 18]. Advances in modern therapy have also led to the use of implants, bioengineered skin substitutes and cell/growth factors to improve efficacy, reduce side effects and improve clinical acceptance [19–21]. Current clinic practice combines both traditional and modern therapies [19]. Epidermal growth factor (EGF) can promote wound healing through its stimulation of the proliferative and migratory capacity of fibroblasts [22, 23]. However, some barriers limit its clinical efficacy, including excessive protease activity and degradation due to chronic infections at the wound site [24, 25].

In this study, to overcome these shortcomings, we combined traditional CUR and modern EGF therapeutics within an aqueous nano-spray. Chitosan was used as a carrier to deliver EGF and CUR because it is a renewable, non-toxic and biodegradable polysaccharide that shows favorable antibacterial and adhesion ability [17, 18]. We fabricated EGF-modified CUR-loaded chitosan nanoparticles (EGF@CCN) to treat full-thickness wounds in *in vivo* Wistar rat models. Curative effects were evaluated through observational and histological analysis of the wound defects.

## Materials and methods

### Materials

Chitosan (CS, *M*w = 30 000, C_6n_H_11n_NO_4n_, deacetylation degree ≥ 95%, viscosity <200 mPa.s) was purchased from Macklin Biochemical Co. Ltd. (Shanghai, China), and Curcumin (CUR) was purchased from Aladdin (Shanghai, China). Sodium tripoly phosphate (TPP) was purchased from the Damao Chemical Agent Factory (Tianjin, China). Phosphate buffered saline (PBS) was purchased from Solarbio Co., Ltd. (Beijing, China). Human Epidermal Growth Factor (EGF) was purchased from Peprotech (Rocky Hill, NJ, USA). All reagents were of analytical grade and used without further purification. Human fibroblast cell lines were purchased from iCell Bioscience Inc. (China, Shanghai). The cells were incubated in a 95% air-humidified atmosphere containing 5% CO_2_ at 37°C.

Thirty healthy Wistar rats (male, 200 g) were purchased from DaShuo Animal Science and Technology Co. Ltd. (Chengdu, China). Rats were housed in specific non-pathogenic conditions at room temperature, at a relative humidity of 50 to 60%, a 12 h light/dark cycle, with free access to standard rodent food and running water. All animals were observed for 1 week prior to experiments, including general conditions such as activity, energy, hair, feces, behavioral patterns and other clinical signs. All animal testing procedures were approved by the ethics and scientific committee of the animal care and treatment committee of the Southwestern Medical University (Luzhou, China).

### Preparation and characterization of CCN

#### Preparation of CCN and EGF@CCN

**Figure 1.**
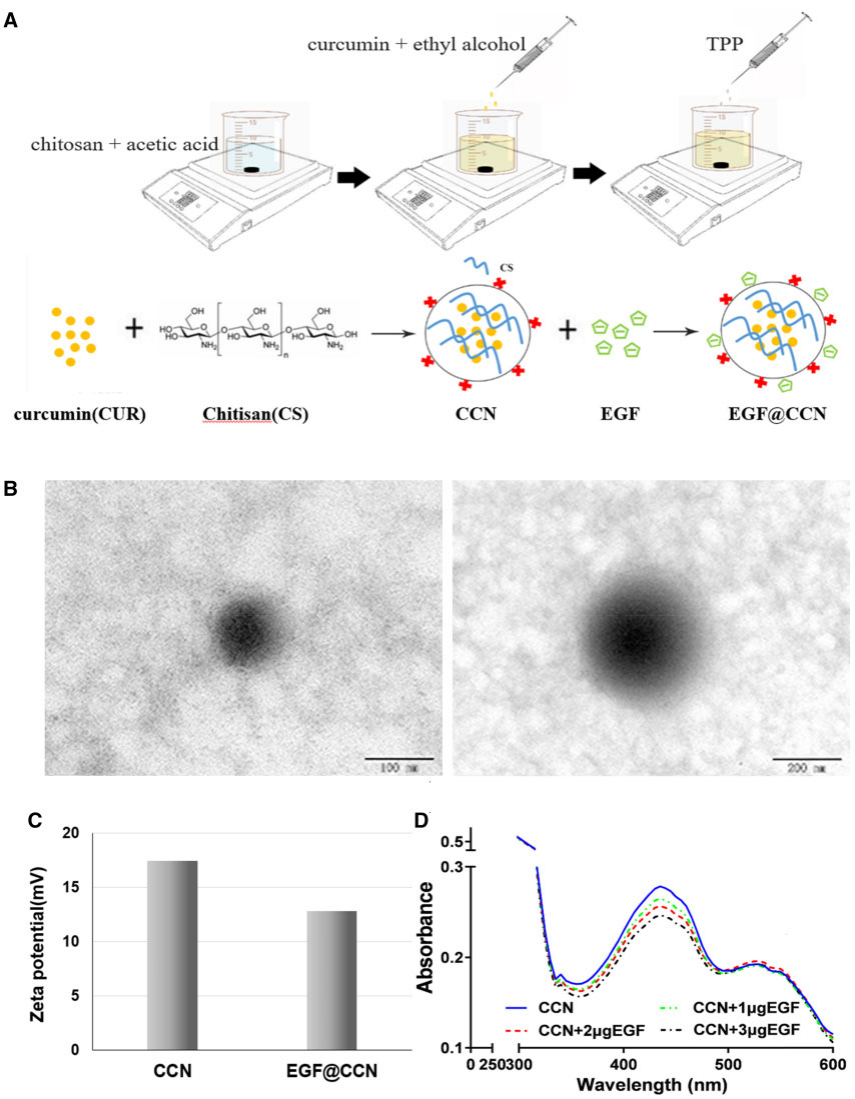
Preparation and characterization of CCN. (**A**) Preparation diagram of CCN; (**B**) TEM image of the CCN; average zeta-potential (**C**) and absorbance (**D**) of CCN and EGF@CCN

CCN were prepared through the ion crosslinking method [26]. Briefly, 25 mg of CS powder was dissolved in 10 ml of 1% aqueous acetic acid (AC), and 10 mg of CUR was completely dissolved in 10 ml anhydrous ethanol (Fig. 1A). The CUR solution was added dropwise into the CS/AC solution under magnetic stirring. Next, 10 mg of TPP was dissolved in 10 ml of water (1 mg/mL) and slowly added to the CUR/CS solution. The mixed solution was stirred at room temperature for 30 min and poured into a dialysis bag with a molecular weight cut-off of 3500 to eliminate residual solvents. After dialysis, the CCN solution was centrifuged at 13 000 r/min for 30 min, and the sediment was washed with water three times and lyophilized. The supernatant was stored at 4°C for further use. Blank chitosan nanoparticles (CS-NPS) were prepared using the same method without the addition of CUR. For preparation of EGF@CCN, 0.2 mg of freeze-dried CCN was dissolved in 1 ml supernatant, followed by ultrasonic treatment at room temperature for 10 min to form a 0.2 mg/mL aqueous solution. Next, 1 µg of EGF was added to 1 ml of CCN solution for 10 min stirring to obtain EGF@CCN. Based on the physics principle of charge neutralization, the negatively charged EGF was absorbed on the positively charged surface of CCN to form EGF@CCN. The absorption spectra of EGF@CCN solutions with different EGF loadings were determined by a UV-VIS absorption spectrometer (UV-5800PC, Yuanxi, Shanghai, China) at room temperature. The resultant nanoparticles were added to a spray bottle prior to use.

#### Characterization of CCN and EGF@CCN

The size distribution and zeta-potential of the produced CCN and EGF@CCN were measured using a NanoBrook 90Plus Zeta instrument (Brookhaven Instruments Corp, Holtsville, NY, USA). The surface morphology of EGF@CCN was investigated using a Tecnai G2 F20 transmission electron microscope (FEI Company, Hillsboro, OR). The entrapment efficiency of CCN was determined through Ultraviolet Spectrophotometry (UV-5800PC, Shanghai, China). The CCN solution was fixed to 10 ml with anhydrous ethanol and centrifuged (12 000 r/min × 40 min). Supernatants were collected and diluted, and the absorbances measured at 425 nm. Finally, the encapsulation rate (EE) and charging efficiency (LE) were calculated as follows:

$$\begin{array}{c} E\mathrm{ntrapment}\;\mathrm{Efficiency}\;\left(EE\right)=\frac{\mathrm{Initial}\;\mathrm{Drug}\;-\;\mathrm{Free}\;\mathrm{Drug}}{\mathrm{Initial}\;\mathrm{Drug}}\times 100\%\\ L\mathrm{oading}\;\mathrm{Efficiency}\;(LE)\;=\frac{\mathrm{Initial}\;\mathrm{Drug}\;-\;\mathrm{Free}\;\mathrm{Drug}}{\mathrm{Total}\;\mathrm{weight}\;of\;\mathrm{NPs}}\times 100\% \end{array}$$

In which, *Initial Drug* represents total amount of added CUR; *Free Drug* represents free amount of CUR in the supernatant.

### *In vitro* drug release

The drug release behavior of each sample was determined using the dialysis method. Free-CUR, CCN and EGF@CCN were dissolved in PBS (pH = 7.4, containing 1% SDS) and prepared as solutions with curcumin concentration of 1 mg/mL. CCN (300 μL), EGF@CCN (300 μL) and free-CUR (600 μL) solutions were, respectively, taken and placed in dialysis bags (*M*w cut-off: 8000). Then the dialysis bags were placed in 30 ml release medium (PBS, pH = 7.4) at 100 r/min, oscillating at a constant temperature of 37°C in a water bath. CCN (1 ml), EGF@CCN (1 ml) and free-CUR (2 ml) releasing medium outside the dialysis bag were collected at pre-determined timepoints and replaced with an equal volume of fresh medium. The absorbance values of each sample were then measured at 425 nm. Due to extremely low concentrations of EGF in EGF@CCN, the absorbance of curcumin will not be affected. The cumulative drug release rate at each time point was calculated.

### *In vitro* cytotoxicity assays

MTT assays were used to determine the *in vitro* cytotoxicity of each sample. Briefly, human fibroblasts were resuspended in DMEM and seeded into 96 well plates at a density of 5.0 × 10^4^ cells per/well. Adhered cells were then treated with CS-NPS, EGF@CS-NPS, free-CUR and CCN for 24 h and 20 µL MTT (5 mg/mL) was added to each well for 4 h. The media was then removed and 150 µL of DMSO was added to each well with shaking for 10 min. Optical density (OD) values were measured at 490 nm once the crystals had dissolved using an iMark microplate absorbance reader (Bio-RAD, USA).

### Antibacterial activity

Two common pathogenic bacteria, *Staphylococcus aureus* (ATCC 29213) and *Escherichia coli* (OP50) were inoculated as scratches onto Agar plates and treated as follows: EGF@CCN, CCN, free-CUR, blank chitosan nanoparticles (CS-NPS) and normal saline (NS). Drug-sensitive paper was infiltrated into the solutions of EGF@CCN, CCN, free-CUR, blank chitosan nanoparticle (CS-NPS) and the NS group, respectively at a concentration of 0.25 mg/mL to prepare the antibacterial tablets for use (diameter = 5 mm). Finally, sterile and dry antibacterial tablets were added to the center of each plate and cultured at 37°C for 24 h prior to imaging. The diameter of antibacterial rings in each plate was measured after 24 h.

### Wound healing assays

Thirty Sprague-Dawley males were selected as models and randomly divided into five groups (*n* = 6): EGF@CCN, CCN, free-CUR, blank Chitosan nanoparticles group (CS-NPS) and NS groups. All animal experiments were approved by the animal protection and use committee and met the relevant national regulations. All animal experiments were approved by the Experimental Animal Ethics Committee of the Southwest Medical University (Luzhou, China).

Asepsis techniques used throughout the study period. Rats were subcutaneously injected with 0.8 ml chloral hydrate at 10% (w/v). Following complete anesthesia, wound defects were established in the loose subcutaneous tissue with a scalpel strip and scissors to a thickness of 2 mm and a diameter of 2.5 cm. During the treatment, we put 2 ml of five different solutions (EGF@CCN, CCN, free-CUR, CS-NPS and NS) into five spray bottles, respectively, and then spray it completely on the wounds. Every 2 ml of drug-loaded solution contained 0.8 mg curcumin and/or 2 μg EGF. In each group, the formulation was sprayed once a day for a total of one week. At each pre-determined timepoint, the wound closure rates were calculated as follows:

$$\mathrm{wound}\;\mathrm{closure}\;\mathrm{rate}\;\left(\%\right)\;=\frac{S^{0}\;-\;S}{S^{0}}\times 100\%$$

*S_0_* and *S* represent the wound area on day 0 and day *t* after surgery.

### Histological analysis

Skin tissue in the wound site was collected from rats in each group, fixed with 10% formalin, paraffin embedded, and sectioned with a Lycra 2500E diamond saw blade (Lycra SpA, Milan, Italy). Sections were stained with hematoxylin & eosin (H&E) and Masson for histological evaluation using light microscopy (Olympus IX73, Tokyo, Japan). The expression of VEGF was detected following immunostaining and imaging of the tissue sections.

### Statistical analysis

Quantitative data are expressed as the mean ± standard deviation (SD). Tukey tests were used for single group comparisons. A one-way ANOVA was used for the analysis of multiple groups. *P*-values ≤ 0.05 were considered statistically significant.

## Results

### Characterization of curcumin-loaded chitosan nanoparticles

In this study, CCN was prepared by ion crosslinking method, and EGF@CCN was successfully prepared by charge neutralization principle as shown in Fig. 1A. We first assessed the morphology and size distribution of the prepared CCN. As shown in Fig. 1B, the monodisperse CCN had a spherical appearance with an average particle size of 210.03 ± 13.48 nm and polydispersity of 0.21 ± 0.005. Entrapment efficiency (EE) and drug loading (DL) were 35.94% and 7.19%, respectively. As shown in Fig. 1C, the average zeta-potential of the CCN were +17.48 ± 0.88 mV. When modified with EGF, the average potential of EGF@CCN decreased to +12.82 ± 0.13 mV. In Fig. 1D, the absorbance of CCN at 425 nm was 0.27 ± 0.001. Following the increase of EGF loading, the absorbance of EGF@CCN decreased gradually, when 3 μg EGF was added to CCN solution, the absorbance value of CCN was 0.24 ± 0.0007. These results highlight the successful preparation of EGF@CCN through charge adsorption between negative EGF and positive CCN.

### *In vitro* drug release and cytotoxicity assessments

**Figure 2.**
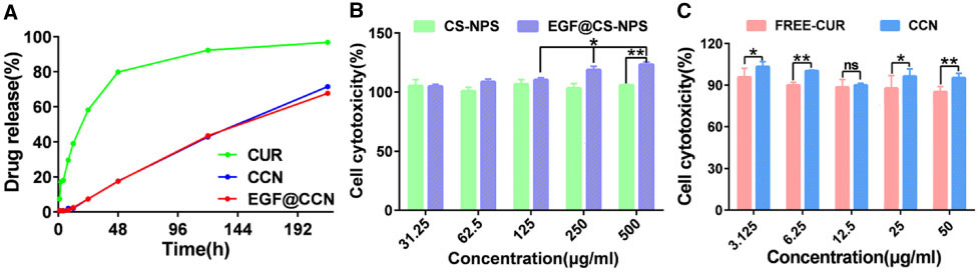
*In vitro* release curves of CUR, CCN and EGF@CCN (**A**); cytotoxicity assay of CS-NPS and EGF@CS-NPS on human fibroblasts (**B**); cytotoxicity assay of FREE-CUR and CCN on human fibroblasts (**C**) (ns: no statistical significance; *P < 0.05, **P < 0.01)

The drug release curves of free-CUR, CCN and EGF@CCN are shown in Fig. 2A. On day 1, ≥ 58% of CUR was released from free-CUR, compared to ∼7% from CCN and EGF@CCN. CCN and EGF@CCN showed a comparable drug release pattern. On day 3, ≥ 93% of CUR was released from free-CUR. On day 9, the cumulative release percentage of CCN and EGF@CCN reached 71% and 68%, respectively. Compared to free-CUR, CCN and EGF@CCN showed more sustained release, with the release from EGF@CCN lower than CCN.

We next investigated the cytotoxicity of CS-NPS and curcumin on human fibroblasts. When the concentration of CS-NPS increased to 500 μg/mL, the survival rates of the fibroblasts remained unchanged (Fig. 2B). It was noteworthy that the proliferative capacity of the fibroblasts increased in response to increasing concentrations of EGF@CS-NPS for 24 h. These data confirm that CS-NPS is a safe and non-toxic carrier, and that EGF@CS-NPS can enhance cell proliferation. In addition, free curcumin showed a tendency of increasing toxicity to human fibroblasts with the increase of drug concentration, while the preparation of CUR coated with nanoparticles (CCN) still presented higher safety with the increase of drug concentration (Fig. 2C). These data confirm that CCN are safer for wound healing.

### Antibacterial activity

**Figure 3.**
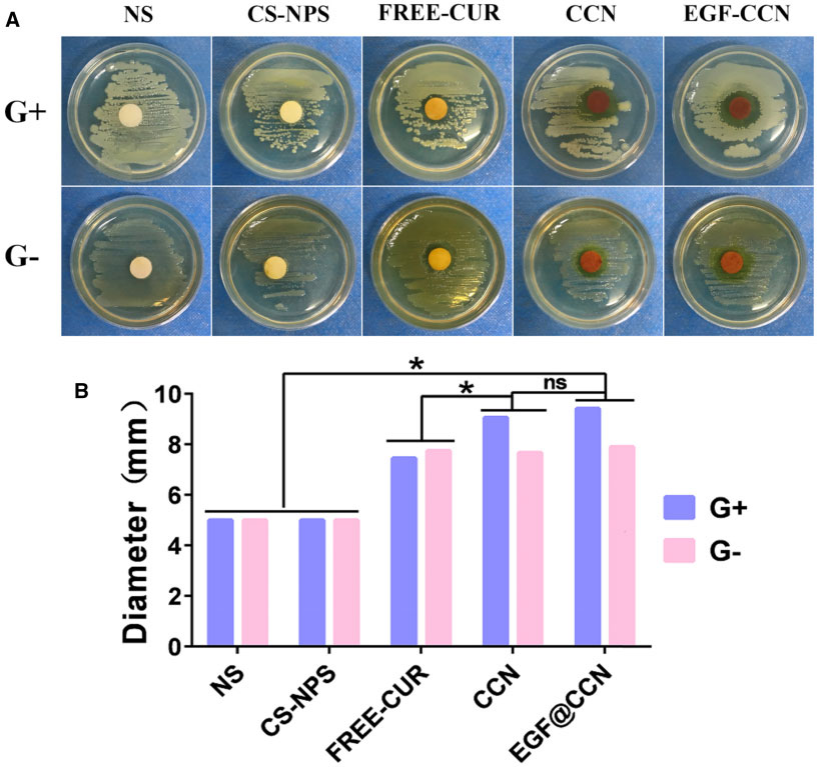
The antibacterial effect of normal saline (NS), CS-NPS, FREE-CUR, CCN, and EGF@CCN on Gram-positive (G+) and Gram-negative bacteria (G−). (**A**) Photos of antibacterial experiments; (**B**) Quantitative analysis of diameter of the antimicrobial rings (ns: no statistical significance; *P < 0.05).

The antibacterial activity of each drug on *S. aureus* (Gram-negative bacteria) and *E. coli* (Gram-positive bacteria) were next investigated. The diameter of the antimicrobial ring was shown in Fig. 3B. In cells treated with EGF@CCN, CCN and free-CUR, obvious bacteriostatic rings against ATCC 29213 and OP50 were observed, highlighting their antibacterial properties due to CUR. The CS-NPS and NS groups showed no inhibition to ATCC29213 and OP50. However, free-CUR led to smaller antibacterial rings than either CCN or EGF@CCN, highlighting the antibacterial activity of CUR. No significant differences were observed between the CCN and EGF-@CCN groups in terms of bacteriostatic rings, proving that the modification of EGF had no negative impact on the antibacterial activity of CCN.

### Wound healing *in vivo*

**Figure 4.**
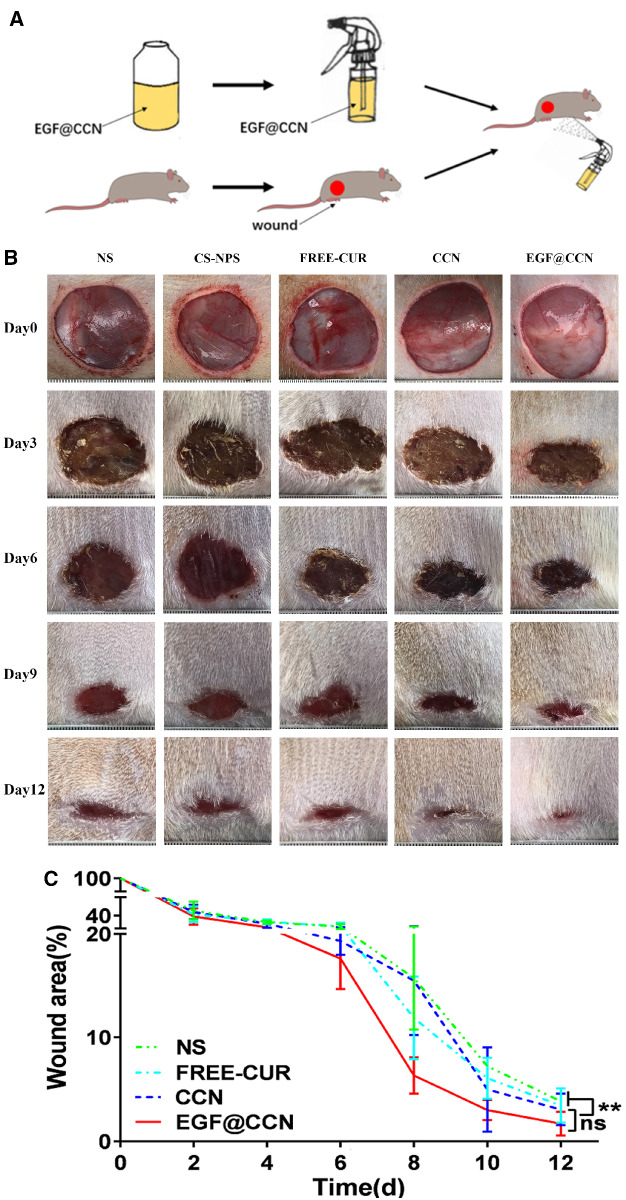
*In vivo* experiment of wound healing on Wistar rats. (**A**) Schematic illustration of experimental procedure; (**B**) Representative pictures of skin wounds treated with normal saline (NS), CS-NPS, Free-CUR, CCN, and EGF@CCN on day 0, 3, 6, 9 and day 12 of post-surgery; (**C**) Quantitative analysis of wound closure rate (%) treated with normal saline (NS), CS-NPS, FREE-CUR, CCN and EGF@CCN at different time points after surgery. (ns: no statistical significance; *P < 0.05, **P < 0.01)

To evaluate the *in vivo* efficacy of the drugs, full-thickness skin defects were established in Wistar rat models. The experimental procedure and wound healing capacity following each treatment is shown in Fig. 4A, B. During the observational period, no redness, exudation, infection, or suppuration occurred in the wounds of the experimental groups. All wounds were covered by blood scabs and showed rapid healing. Rats treated with EGF@CCN showed faster wound healing. On the 12th post-operative day, wounds in the EGF@CCN group had healed, with wound healing rates as high as 98.04%, higher than that of the free-CUR (96.56%) or NS group (96.16%). Moreover, there are statistically significant differences between group EGF@CCN and Group NS, group EGF@CCN and Group CS-NPS on the 12th day (*P* < 0.05).

### Histological analysis

**Figure 5.**
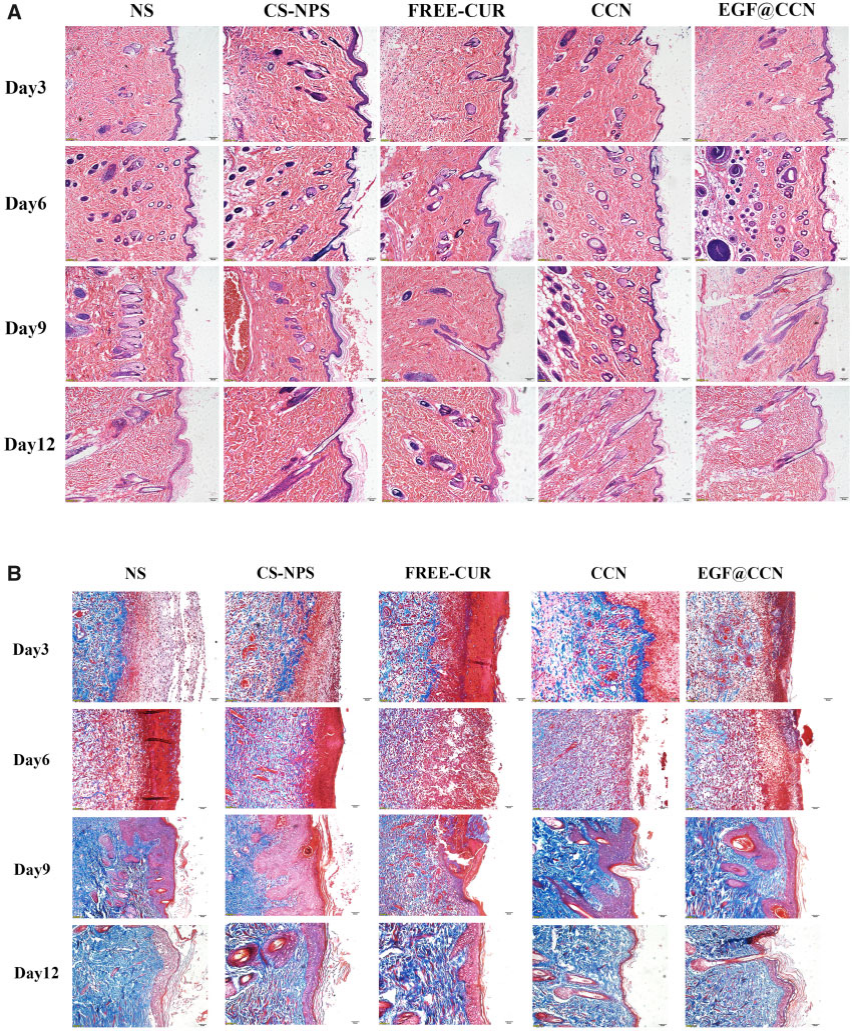
Representative images of H&E (**A**) and Masson staining (**B**) of skin wounds treated with normal saline (NS), CS-NPS, FREE-CUR, CCN and EGF@CCN on day 3, 6, 9 and day 12 post-treatment. (A) scale bar = 10 μm, magnification 100×. (B) scale bar = 5 μm, magnification 200×

**Figure 6.**
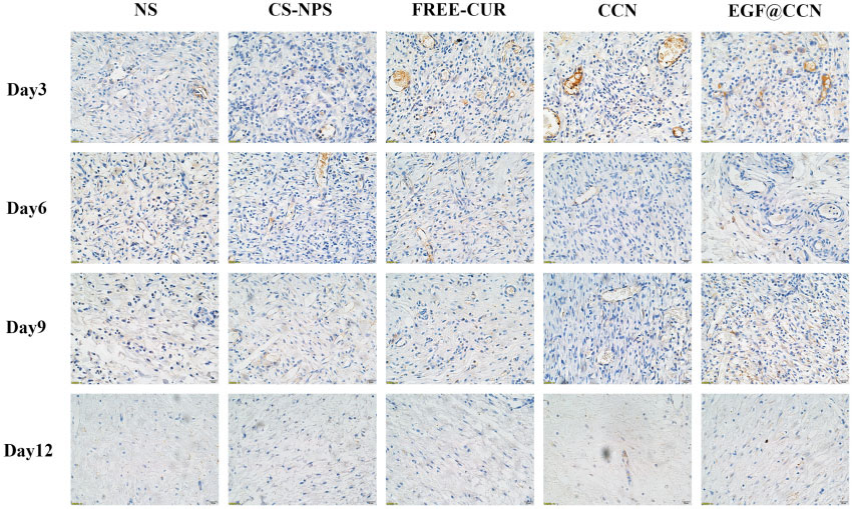
Fluorescent images of VEGF staining of skin wounds treated with normal saline (NS), CS-NPS, FREE-CUR, CCN, and EGF@CCN on day 3, 6, 9 and day 12 of post-treatment. Scale bar = 2.5 μm, magnification 400×

H&E stained images of the wounds are shown in Fig. 5A. On the 1st post-operative day, inflammatory cell infiltration and the formation of granulation tissue could be observed in all groups. However, compared to NS, free-CUR, and CS-NPS, wounds treated with CCN and EGF@CCN showed fewer inflammatory cells and the presence of fibroblasts was more obvious, indicating that the inflammation was inhibited. On days 6, 9 and 12, the CCN and EGF@CCN groups showed significantly fewer inflammatory cells, a more regular arrangement of collagen fibers, and more obvious epithelial hyperplasia keratosis than the control group. Moreover, the EGF@CCN group showed the most significant reduction in inflammatory cell numbers, with epithelial keratosis and hair follicle formation comparable to normal skin. Fig. 5B shows the deposition of collagen through Masson staining (purple represents the deposition of collagen). On day 9, collagen deposition in the CCN group and EGF@CCN groups were higher than those of other groups. On day 12, collagen deposition in the CCN and EGF@CCN groups was more compact and mature. In the NS, free-CUR, and CS-NPS groups, a large degree of inflammatory infiltration on days 6 and 12 were observed. Figure 6 shows VEGF staining of the wounds on days 3 and 9, in which neovascularization in the CCN and EGF@CCN groups were higher than those of the other three groups. The highest levels of angiogenesis were observed in the EGF@CCN group.

## Discussion

Wound dressing is key to the maintenance of a moist environment that promotes wound healing [27]. The micro-environment can enhance the migration of fibroblasts and epidermal cells during wound healing. Skin dressings are available in a variety of forms, including fibrous membranes and gels. The dressing matrix includes synthetic polymers such as PLA, PEG-PCL and PLGA, and natural polymer materials including chitosan, sodium alginate, hyaluronic acid, cellulose and proteins [28]. During the preparation of wound dressings, various drugs and/or growth factors can be incorporated into the matrix to enhance the healing functions of the dressing [29]. Due to its excellent expansion ability and high biocompatibility, chitosan has attracted intense research attention as a component of skin dressings [17]. In addition, curcumin (CUR) is frequently used as a therapeutic agent in wound repair, owing to its excellent antibacterial and anti-inflammatory properties.

Based on the positive role of CUR and chitosan in promoting skin repair, we prepared CUR-loaded chitosan nanoparticles (CCN) and modified CCN with EGF to obtain an EGF@CCN nano-spray. EGF is a small polypeptide with a molecular weight of ∼6 kDa that shows robust skin repair function that can accelerate wound healing by enhancing the composition of the basement membrane and extracellular matrix, increasing cell migration and proliferation [30, 31]. In addition, in terms of drug delivery, the commonly used fibrous mats are difficult to fix onto wound sites, and often fail to meet the shape of irregular wounds. Although soft hydrogels can cover irregular wounds, their poor permeability affects the exchange of nutrients such as oxygen during skin repair [32]. Compared to conventional wound dressings, the advantages of the spray include its convenience of storage, ease of preparation, and ability to fit a range of irregular wounds with high adhesion ability [33]. We therefore designed EGF@CCN as a spray-delivery system for the simultaneous delivery of CUR and EGF.

When wounds are exposed to air, the risk of bacterial infection can prevent their healing, leading to greater damage. Skin dressings should therefore retain antibacterial activity to prevent the proliferation of bacteria in the wound site [34, 35]. A large number of studies have shown that this function can be achieved through the addition of antimicrobial agents into the dressings [35]. In this study, we confirmed that the modification of CCN with EGF did not weaken the antibacterial activity of CUR on both *S. aureus* (Gram-negative bacteria) and *E. coli* (Gram-positive bacteria). In addition to their antibacterial function, the biocompatibility of the skin dressings during wound reconstruction are of high importance [36]. Only dressings that are non-toxic to cells and tissues are applicable to clinical practice. We showed that CS-NPS causes no toxicity to normal fibroblasts within a concentration range of 0–500 μg/mL, CCN is also safer than free-CUR. More importantly, we found that the EGF-modified CS-NPS showed excellent proliferative effects on normal fibroblasts at all concentrations assessed. Taken together, these data suggest that the developed nano-spray is safe, non-toxic and promotes wound repair [37]. *In vitro* drug release analysis showed that CUR was slowly released from CCN and EGF@CCN.

The effects of the developed EGF@CCN nano-spray on skin defects in rats confirmed a high efficacy to promote wound healing. On day 12, the rats treated with EGF@CCN had an average wound closure rate of ∼98%, which was higher than all other groups. Histological analysis also confirmed changes in VEGF expression (Figs 5 and 6). Rats treated with EGF@CCN showed thickened epithelial keratosis, reduced inflammatory cell infiltration and more rapid neovascularization. Collagen deposition was also observed during wound healing and increased over time. In summary, EGF@CCN not only maintained the biological activity of CUR and the strong repair effects of EGF, but when used with a convenient spray, effectively accelerated wound healing within the skin defects. This therefore represents a promising therapeutic agent for wound repair.

## Conclusions

We developed an EGF-modified CUR-loaded chitosan nano-spray (EGF@CCN) as a wound healing agent to enhance the healing of dermal defects in rat models. Both blank chitosan nanoparticles (CS-NPS) and EGF@CS-NPS showed excellent cyto-compatibility in human fibroblasts, revealing the prepared carrier as safe and non-toxic. EGF@CCN showed antibacterial activity and a slow drug release pattern *in vitro*. Full-thickness dermal defects in rats treated with EGF@CCN showed higher wound healing rates, improved bacteriostatic effects, higher levels of angiogenesis, reduced inflammatory cell infiltration and increased collagen deposition, ultimately accelerating re-epithelialization. As such, the EGF@CCN nano-spray can improve wound healing and the treatment of skin wounds caused by tumor resection, diabetes, and accidental injuries.

## Funding

This work was supported by the Project Program of the Science and Technology Department of Sichuan Province (2020YJ0385), the Union Project of Luzhou Municipal People’s Government-Southwest Medical University (2018LZXNYD-ZK06, 2019LZXNYDJ46), the Projects of Sichuan Medical Association (Q17080).

*Conflict of interest statement.* None declared.
